# “Every Time It Comes Time for Another Shot, It’s a Re-Evaluation”: A Qualitative Study of Intent to Receive COVID-19 Boosters among Parents Who Were Hesitant Adopters of the COVID-19 Vaccine

**DOI:** 10.3390/vaccines12020171

**Published:** 2024-02-07

**Authors:** Ramey Moore, Rachel S. Purvis, Don E. Willis, Ji Li, Jonathan Langner, Morgan Gurel-Headley, Shashank Kraleti, Geoffrey M. Curran, Michael D. Macechko, Pearl A. McElfish

**Affiliations:** 1 College of Medicine, University of Arkansas for Medical Sciences Northwest, 2708 S. 48th St., Springdale, AR 72762, USA; rameymoore@uams.edu (R.M.); rspurvis@uams.edu (R.S.P.); dewillis@uams.edu (D.E.W.); 2 Fay W. Boozman College of Public Health, University of Arkansas for Medical Sciences Northwest, 2708 S. 48th St., Springdale, AR 72762, USA; 3 Office of Community Health and Research, University of Arkansas for Medical Sciences Northwest, 2708 S. 48th St., Springdale, AR 72762, USA; jlangner@uams.edu; 4 College of Medicine, University of Arkansas for Medical Sciences, 4301 W. Markham St., Little Rock, AR 72205, USA; mpgurel@uams.edu (M.G.-H.); skraleti@uams.edu (S.K.); 5 Fay W. Boozman College of Public Health, University of Arkansas for Medical Sciences, 4301 W. Markham St., Little Rock, AR 72205, USA; 6 College of Pharmacy, University of Arkansas for Medical Sciences, 4301 W. Markham St., Little Rock, AR 72205, USA; currangeoffreym@uams.edu; 7 Center for Mental Healthcare and Outcomes Research, Central Arkansas Veterans Healthcare System, 4300 W. 7th St., North Little Rock, AR 72114, USA; 8 College of Medicine, University of Arkansas for Medical Sciences Northwest, 1125 N. College Ave., Fayetteville, AR 72703, USA; mdmacechko@uams.edu

**Keywords:** vaccine hesitancy, COVID-19 vaccine booster dose acceptance, vaccine decision making

## Abstract

COVID-19 vaccine coverage remains low for US children, especially among those living in rural areas and the Southern/Southeastern US. As of 12 September 2023, the CDC recommended bivalent booster doses for everyone 6 months and older. Emerging research has shown an individual may be vaccine hesitant and also choose to receive a vaccine for themselves or their child(ren); however, little is known regarding how hesitant adopters evaluate COVID-19 booster vaccinations. We used an exploratory qualitative descriptive study design and conducted individual interviews with COVID-19 vaccine-hesitant adopter parents (n = 20) to explore COVID-19 parental intentions to have children receive COVID-19 boosters. Three primary themes emerged during the analysis: risk, confidence, and intent, with risk assessments from COVID-19 and COVID-19 vaccine confidence often related to an individual parent’s intent to vaccinate. We also found links among individuals with persistent concerns about the COVID-19 vaccine and low COVID-19 vaccine confidence with conditional and/or low/no intent and refusal to receive recommended boosters for children. Our findings suggest that healthcare providers and public health officials should continue making strong recommendations for vaccines, continue to address parental concerns, and provide strong evidence for vaccine safety and efficacy even among the vaccinated.

## 1. Introduction

Infection with COVID-19 can result in severe outcomes in children, including hospitalization and death [1,2,3]. Children can also spread COVID-19 infection even when asymptomatic or presenting mild symptoms [2]. However, COVID-19 vaccine coverage remains low among the pediatric population [4,5]. As of May 2023, only approximately 44% of children aged 6 months to 17 years had received the first dose of the COVID-19 vaccine in the United States (US) [6]. Data from the National Immunization Survey—Child COVID Module show that only 18.5% of US adolescents 12–17 years who completed the primary series have received a dose of the bivalent booster [7]. US adolescents residing in rural areas had reduced COVID-19 vaccine coverage compared to those in urban areas, with only 6.9% being up-to-date with COVID-19 vaccination compared to 11.9% in major US cities [7]. COVID-19 vaccine coverage in the Southern/Southeastern US has lagged compared to the rest of the country, and at the end of 2022, Arkansas ranked 44th in the country for children vaccinated against COVID-19 [8]. Vaccination remains a major strategy for preventing severe COVID-19 in children [9]. Thus, it is critical to understand drivers of low vaccination rates among children and adolescents, such as parental vaccine hesitancy, to mitigate the spread and severity of COVID-19 infections, especially in areas where COVID-19 vaccination coverage is already lacking [3].

Vaccine hesitancy remains an important global public health concern, with some indications that hesitancy towards vaccination is increasing in scope and scale [10,11,12,13]. We define vaccine hesitancy as an attitude or orientation to avoid conflating vaccine hesitancy with vaccination behaviors [10,14,15,16]. Emerging research has shown that an individual may be vaccine hesitant and also choose to receive a vaccine [10,14,15,17,18,19,20,21,22,23,24,25,26]. These individuals are hesitant adopters [15,17,27]. An examination of hesitant adopters could improve our understanding of how vaccine hesitancy affects future vaccine decision making [28,29]. A recent quantitative study demonstrated that vaccine hesitancy affected the uptake of COVID-19 boosters even among those who had received prior vaccinations [30]. A small body of literature has focused on vaccination as an ongoing process, where an individual’s orientation towards vaccination may not be fixed, but instead is dynamically related to the social and health contexts for vaccine decisions [28,31,32,33,34]. Hesitant adopters are an important population for further research to understand the dynamic, ongoing effects of vaccine hesitancy on vaccine uptake.

As of 12 September 2023, the Centers for Disease Control and Prevention has recommended bivalent booster doses for everyone 6 months and older, which may mitigate waning immunity and protect against new COVID-19 variants [35], and further boosters may be necessary to mitigate waning immunity, protect against new COVID-19 variants, and protect against severe disease [9,29,36,37,38]. Significant gaps remain in our understanding of the relationship among parental vaccine hesitancy, prior vaccination behavior, and intention to have children receive a future COVID-19 booster. To the authors’ knowledge, no previous qualitative studies have explored parental intention to have children receive COVID-19 booster doses among hesitant adopters. The objective of the present study is to explore hesitant adopter parents’ intentions to have their child(ren) receive COVID-19 boosters.

## 2. Methods

### 2.1. Study Aims and Procedures

The study aimed to understand parental COVID-19 vaccine hesitancy and parents’ intention to have their children receive COVID-19 vaccine boosters. We used an exploratory qualitative descriptive design drawing on individual interviews with parents who were hesitant adopters of the COVID-19 vaccine (n = 20), with a complementary analysis of survey data [39,40]. All study materials and procedures were reviewed and approved by the Institutional Review Board of the University of Arkansas for Medical Sciences (IRB#274483).

### 2.2. Participant Recruitment and Study Sample

The research team identified potential participants from a prior phone survey of 2201 adults, the results of which are published elsewhere [30,41]. Survey participants were asked if they were willing to be contacted for a qualitative interview. The original survey was conducted in Arkansas in October 2022. In addition to sociodemographic information, participants were asked to report the extent to which they were hesitant about receiving the COVID-19 vaccine. Inclusion criteria for qualitative interviews included adults (≥18 years old), Arkansas residents, reporting any level of COVID-19 vaccine hesitancy, having at least one child vaccinated against COVID-19, and consenting to be contacted for a follow-up interview. A total of 110 potential participants met the inclusion criteria. The research team contacted potential participants via email to request an interview. A total of 20 participants responded affirmatively and completed qualitative interviews. These individuals comprise the final qualitative sample. All participants were provided study information via email, and interviewers reviewed study information with participants and allowed time for them to ask questions before interviews. Qualitative interview participants provided verbal consent and received a $50 gift card as remuneration upon completion of the interview.

### 2.3. Data Collection

Interviews were conducted between November 2022 and January 2023 by three members of the research team with qualitative interview expertise. Participants received unique meeting invitations from the research team via email, and interviewers were assigned based on availability. To ensure consistency across interviews, we developed a semi-structured interview guide, using an iterative process [42,43]. The research team revised the interview guide three times before conducting interviews. The interview guide consisted of questions asking participants to report their thoughts and feelings about vaccines, specifically about their attitudes toward the COVID-19 booster and their intention to have their child(ren) receive future COVID-19 booster doses. All interviews were conducted over Zoom and lasted between 25 and 60 min. Interviews were recorded and transcribed verbatim by a professional transcription service.

Demographic information was collected using survey items adapted from the Behavioral Risk Factor Surveillance System (BRFSS) [44]. We used a vaccine hesitancy question adapted from Quinn and colleagues to collect self-reported levels of COVID-19 vaccine hesitancy [20]. Participants could indicate whether they were “very hesitant”, “somewhat hesitant”, “a little hesitant”, or “not at all hesitant”. Participants were asked to report their trust in the COVID-19 vaccine, using a Likert scale with the options “very”, “somewhat”, “a little”, and “not at all”. To collect the vaccination status of participants’ child(ren), we asked, “Have all, some, or none of your children who are between 6 months and 17 years old been vaccinated against COVID-19?” Response options included “yes, all of them”, “yes, some but not all of them”, and “none of them”. Participants were asked about current or prior COVID-19 infection, using the question, “To your knowledge, do you have or have you had COVID-19?” All parents were asked about their child(ren)’s current or prior infection with COVID-19 with the question, “Have any of your children tested positive for, or do you suspect any of your children have had, COVID-19”? Response options included “yes, one or more tested positive”, “yes, I suspect 1 or more have had COVID-19”, “no”, or “don’t know/not sure”. We used the question, “Has a close friend or relative died of COVID-19?” to collect COVID-19 death exposure.

### 2.4. Data Analysis

The research team calculated descriptive statistics (frequencies and percentages) of the sociodemographic characteristics for the qualitative samples, which we present below. We calculated participants’ self-reported COVID-19 vaccine trust, level of vaccine hesitancy, past and current COVID-19 infection for the participant and their child(ren), and COVID-19 death exposure with qualitative themes to provide context and confirmation of qualitative themes. These are reported below and described with the appropriate primary themes or subtheme(s). All interview transcripts were imported into a password-protected MAXQDA 2020 project file to facilitate interpretation of the data, identification of patterns, and labeling of data segments with emergent secondary codes [45]. The first author and two co-authors used a thematic analysis approach to identify and refine codes. The first author conducted initial coding and developed a preliminary codebook. We revised the codebook three times, using a consensus model to resolve all coding discrepancies. Using a constant comparative approach where each datum is compared and contrasted with all other data in an iterative process, two co-authors conducted confirmation coding of all transcripts. This process ensured the rigor, reliability, and trustworthiness of the analysis. A heatmap of co-occurring codes and a cluster plot to understand associations among emergent codes and themes was generated using MAXQDA 2020, with codes considered to be co-occurring if appearing within three data segments (i.e., single statements or short collections of related statements within interview transcripts) [45,46]. The research team critically reviewed the data, coded data segments, and the final codebook and selected exemplary quotes that best represented emergent themes.

## 3. Results

We present the demographics of study participants (n = 20) in Table 1. Participants were all hesitant adopter parents with at least one child who had received the COVID-19 vaccine. Participants’ ages ranged from 30 to 73 years, with a mean age of approximately 43 years. More than half of the participants were women (65.0%). The sample was diverse: 65.0% self-identified their race/ethnicity as White, 20.0% as Black/African American, 10.0% as Hispanic, and one (5.0%) as multiracial/other. Most participants reported their relationship status as married (65.0%), with the rest reported as divorced (15.0%), widowed (5.0%), or never married (15.0%). Participants self-reported their level of education as high school graduate or equivalent (20.0%), some college, no degree (10.0%), associate degree (20.0%), bachelor’s degree (25.0%), and graduate degree (25.0%). The majority (95.0%) reported current healthcare coverage. Most (75%) participants indicated that all of their eligible children were fully vaccinated against COVID-19. Slightly over half of participants described themselves as “a little hesitant” (55%), and slightly less than half described themselves as “very hesitant” (40%) about the COVID-19 vaccine. Participants reported their level of trust in the COVID-19 vaccine as “not at all” (10%), “a little” (25%), “somewhat” (25%), and “very much” (40%). As context for risk assessments from COVID-19 infection, participants were also asked whether they have had, or currently had, COVID-19, with most responding affirmatively (65%). Participants were also asked whether their child(ren) had ever tested positive, or if they suspected that they had had COVID-19, with most indicating “yes, tested positive” (75%), 10% responding “yes, suspected”, and only 15% reporting “no”. To provide further context for participant perceptions of risk from COVID-19 infection, we collected exposure to death from COVID-19, with most hesitant adopter parents reporting that they have not had a close friend or relative die from COVID-19 (65%).

Three primary themes emerged when participants discussed their perceptions of the COVID-19 vaccine and boosters: risk, confidence, and intent. Within the primary theme of risk, we identified two emergent subthemes: “reduced risk from COVID-19 infection” and “vaccine/booster is risky”. For the primary theme of confidence, two subthemes emerged: “low confidence” and “high confidence”. Within the primary theme of intent, the analysis highlighted three subthemes: “strong intent”, “conditional intent”, and “low/no intent”. Primary themes and subthemes are collected in Table 2.

### 3.1. Risk

Hesitant adopter parents described balancing perceived risks from COVID-19 infection against perceived risks from the COVID-19 vaccine. Additionally, many participants described their own and/or their child(ren)’s mild experiences with COVID-19 infection as an influence on their fear of COVID-19 infection, which was often balanced against perceived risks from the COVID-19 vaccine.

*Reduced risk from COVID-19 infection.* Many participants felt the current health risks from COVID-19 infection were low or significantly reduced: “Its potency, you know, COVID is—has gotten weaker and weaker that at this point, its—well, you know what’s the point?” (48241424). Another father identified the lack of news coverage and discussion of COVID-19 in his local community as an important factor in estimating the dangers of COVID-19 infection. He stated, “They really don’t bring it up anymore since, you know what I’m saying, it’s not across the news and [they don’t] make you wear the mask”, which affected his understanding of the current risks and imperative to have children in his home receive boosters (43575619). Other participants linked reduced perceived risk from COVID-19 to being in overall good health, often noting a lack of co-morbidities or underlying conditions that might increase individual risk. One participant succinctly stated, “I just felt like, we’re pretty—we’re healthy people. Um, you know, I’m like, ‘We can just skip the booster’” (45260584). Others focused on perceived immunity or protection from their current amount of vaccination, past COVID-19 infections, or a combination of the two. One participant stated, “We decided to stop because we got it [COVID-19], and we feel like we’re protected enough at this point”. This participant stated that she received an initial third shot booster: “Then we actually got COVID last January, a year ago. And so, after that we figured we’ve had three shots and the COVID, it doesn’t seem like it’s as big of a dangerous risk at this point for us” (52331974). Another hesitant adopter parent described their perception of decreased risk from COVID-19 infection affecting their children: “I just think that their body and their abilities to fight it, they’ve been around people. They’ve been exposed, and they have not contracted it. So, I think it’s not as big of a deal as it was then” (48030030).

*Vaccine/booster is risky.* Hesitant adopter parents described perceived risks related to COVID-19 vaccines and booster doses, often connected to lingering feelings of concern, hesitancy, and perceived risks from vaccines. One hesitant adopter mother, who reported working in healthcare during the COVID-19 pandemic, contrasted the COVID-19 vaccine with influenza vaccines. She stated, “The flu shot has been around long enough and studied long enough that I feel comfortable”, contrasted with “seeing problems […] related to that [COVID-19] vaccine I feel like it was just put out there too quick” (45260584). Other participants also linked the speed of development and testing of the vaccine as a source for their hesitancy. One participant phrased this as a question, “How often was this tested out? Like, did they just come out with this, or is it even safe to consume?” (50244610). Another hesitant adopter parent highlighted side effects and adverse events cataloged in the US Vaccine Adverse Event Reporting System (VAERS). He stated, “VAERS now is up to almost 7000 deaths. All of them almost are younger people, I think there’s been more VAERS reported, deaths after the COVID vaccine than there are people in those age cohorts that have died of COVID” (48134839).

Side effects were frequently mentioned as a factor in parental risk perceptions: “For my kids, I was like, they might have a reaction to [the COVID-19 vaccine] or something of that nature or whether they might get real sick” (51695305). Other hesitant adopter parents focused on the unknown effects of taking the vaccine. As one participant stated, “I mean, it’s a pretty big deal to be, like, pumpin’ people full of unknowns” (48567255). Another parent stated, “Maybe I’m overthinking it, *[laughter]* but I don’t want extra dosages. I mean, we wanna do what we need to do to protect our kids, but I don’t really wanna line them up and get two doses if one’ll provide them the same protection” (49632146). One hesitant adopter mother, who works in an obstetrics and gynecology practice, described regretting her decision to have her daughter vaccinated. This participant attributed her regret to menstruation- and fertility-related side effects she observed in patients at the clinic in which she worked. She described her fears of these side effects and perceived risks from further vaccination as being elevated, while on the other hand, she felt the risks of COVID-19 infection were lowered for her “healthy 16-year-old [daughter] with no co-morbidities” (49632146). Participants frequently balanced perceived risks from COVID-19 infection with risks from the COVID-19 vaccines. One hesitant adopter mother explained, “By the time the boosters started becoming available or recommended […] I didn’t feel like at that point the risk of the unknown of the vaccine outweighed the risk of the infection itself” for her daughter (42664156). A hesitant adopter father stated that he did not “plan on pursuing” booster shots for his son, stating, “I think he got sicker from [the vaccine] than when he got sicker from COVID” (48241424). This participant described his evaluation of the risks of infection versus the vaccine:

If let’s say, 30 out of 100 people that got COVID, you know, were to die or have, you know, severe issues, then, that’d be one thing. [But,] when it came to kids, it wasn’t even one out of 100, it was like point something out of 100 or something, you know. And then you hear the stories about the heart issues and the myocarditis […] for [a disease] that didn’t really have a huge downside, what’s the point? Why run the risk?

(48241424).

### 3.2. Confidence

Hesitant adopter parents frequently explored their confidence in COVID-19 vaccines and boosters, including both “low confidence” and “high confidence” in the vaccine or boosters. As we report above, most participants expressed some level of reduced trust in the COVID-19 vaccine, with only 40% stating that they “very much” trust the COVID-19 vaccine. Many participants directly related initial hesitancy to low confidence in the COVID-19 vaccine, with these prior concerns remaining salient for their attitudes and planned intent to have a child(ren) receive COVID-19 boosters.

*Low confidence.* Many participants articulated low levels of confidence in the COVID-19 vaccine and boosters. One hesitant adopter mother stated, “I feel like Pfizer was not, they weren’t completely honest about what was goin’ on. And […] after reading some of the things, afterwards, you know, I was shocked that they didn’t disclose that to the public” (45260584). While all participants described themselves as hesitant about the COVID-19 vaccine, some hesitant adopter parents described their trust and confidence in the COVID-19 vaccine as decreasing throughout the COVID-19 pandemic: “Prior to the [COVID-19] jabs, I was pretty vaccine positive” (48134839). This participant continued that he would “never get another one” for himself or his children. He explained this drastic negative change in his confidence due to “the lies” told by “the government” concerning the risks of myocarditis and false claims concerning COVID-19 vaccine efficacy to prevent transmission. From this, he concluded, “Then it comes out, well, we never even tested it for transmission, which means I put something that hadn’t been fully tested into my body and into my kid’s bodies”. This participant also linked his low confidence to politicized public health messaging, stating the following:

Some of the people that we should trust no matter what, um, you know, are the people in charge of medical disasters, you know, disease control, [and] you trust these people […] instead, they’d rather just get their 15 min of fame, and they pushed the narrative of whoever’s in charge, because if they don’t, they’re not gonna get airtime, they’re gonna get fired and everything else

(48134839).

*High confidence.* Some hesitant adopter parents reported “high confidence” in the COVID-19 vaccine. One hesitant adopter mother described the feeling of her daughter being fully vaccinated and having received a booster: “The moment I thought about the vaccine was like a relief for me, honestly. […] Honestly, for me, the fact of receiving the vaccine in the children, in my mother who has cancer, in me, it was like a relief for me. To feel protected; yes, protected” (51145138). One participant described herself as “most definitely” wanting to receive a yearly booster, because “in the beginning when I didn’t have the shot, when we contracted COVID it was bad, but afterwards, even though I was exposed to COVID, people with COVID, I felt like my symptoms were lesser” (50244610).

### 3.3. Intent

Hesitant adopter parents presented varied intent for future decisions to accept COVID-19 vaccines for their children: “strong intent”, “conditional intent”, and “low/no intent”. Hesitant adopter parents linked their intentions to accept further doses of the COVID-19 vaccine for their children to their perceptions of risk and their confidence in the vaccine. Strong intent was associated with high confidence, with conditional and low/no intent associated with greater perceived risk from the COVID-19 vaccine/booster and, to a lesser extent, perceptions of reduced severity of or danger from COVID-19 infection. One hesitant adopter mother described herself and her family as “generally trusting of that system and that process” for testing and approving vaccines (49632146). However, this participant also emphasized the dynamic nature of confidence for her children receiving future boosters, stating, “It’s an ongoing conversation at this point. […] Every time it comes time for another shot or time for a booster, it’s a reevaluation of the risk of the shot and the timing. You know, it’s the, well, you’re eligible for one, but does that mean that today is the day to get it?”

*Strong intent.* Some hesitant adopter parents described a strong intent to receive boosters for their children, describing the vaccine as a normal part of their health-seeking behavior for their children. One participant stated, “Now [the COVID-19 vaccine] is something like natural, normal. I only think, ‘Oh, the time to take our vaccine is coming.’ And, in my house, all of us take it when it’s time to-since we have different schedules; if I don’t have to work, first two of us, then three, and like that, to be vaccinated but it’s a normal thing now” (51145138). This participant articulated another key factor in her intended acceptance of COVID-19 vaccine boosters for her children: “Just, like, pneumonia vaccines when people are due for it again, just like a tetanus shot only ten years. I think it’s not gonna last in your body forever, so you’ll have to re—it’s kinda like a maintenance, like an oil change” (51145138). Another mother articulated a strong intent to have her child receive further booster doses of the COVID-19 vaccine, linking intent to the evaluation of risk and confidence in vaccine efficacy. She stated, “I’m in favor of every vaccination I can get my child”, and continued, “Anything I can do to make sure that, you know, it won’t turn into something more serious I am highly in favor for” (48018934).

*Conditional intent.* Many participants described conditional intent to have children receive boosters, for example, articulating conditions related to increased future risk from COVID-19 infection, emerging research on specific topics, and receiving a recommendation from a trusted source. One hesitant adopter mother stated that she would consider future booster doses based on her evaluation of risk:

How the spread of COVID is going through, like, the winter times, like flu? Is it gonna be ramped up and huge again where we see that? Okay, maybe we need a seasonal COVID vaccine, maybe we need a booster. But if we’re not seeing that widespread population getting COVID. I don’t really feel like it should be that we get a vaccine all the time for it.

(48030030)

Another hesitant adopter mother described her intention as conditional on further research related to the risk on reproductive health: “I think specifically if there was proven research that it was not affecting fertility […] just because of […] the irregular bleeding and things like that that I have personally seen in the clinic” (48030030). She continued that this research was “the only thing that would reassure me I think”.

Some participants identified specific trusted sources of information that would improve their confidence in the COVID-19 vaccine and ultimately drive their conditional acceptance. One parent stated, “I have a lot of faith in one of our medical directors […] and if he were to say I’m going to get my kids boostered, I would probably weigh heavily on that and probably do the same” (48030030). Importantly, statements of conditional acceptance sometimes included more than one salient factor, such as specific additional research results from a trusted pediatrician. As one participant stated, “If I’ve got the facts in front of me [from my daughter’s pediatrician], then I feel like I can make a better-educated decision […] And, yeah, I do trust my pediatrician and I do value their opinion” (48567255).

*Low/no intent*. Hesitant adopter parents who described low/no intent to accept COVID-19 boosters frequently mentioned low perceived risk from COVID-19 infection. As one parent said, “We’re healthy people. You know, I’m like, ‘We can just skip the booster’” (45260584). Another participant stated the following:

There’s probably still people that die from [COVID-19], but, you gotta look at their core morbidities, […] they’re probably obese, they probably have diabetes, they probably have a chronic respiratory issue, [and] if you really go back and do the math, you know, COVID probably didn’t actually, kill that many people, […] it’s kinda like AIDS, you know, nobody dies of AIDS, they die of what AIDS does to you.

(48241424)

Other participants cited the perceived risk that the vaccine and its side effects posed as affecting their lack of intent. One participant stated, “I won’t take the booster. My husband did. I won’t. My kids won’t. But the side effects of the vaccine were awful. It was worse than having COVID, and I had COVID. […] It’s not worth it. I’m not gonna put my kids through somethin’ like that” (45260584).

Several hesitant adopter parents also linked prior motivation to vaccinate to mandates, which have since been lifted. These participants stated that, without the mandate, they do not intend to get future COVID-19 vaccines. As one participant explained:

Me and my husband got the booster shot but the children they haven’t got yet, and the reason why is because once they got their first and second dosages, the mandate was lifted so, they were capable of, you know, going back to school and, just kinda resuming life a little bit easier without the pressure of getting that booster shot.

(50244610)

Another hesitant adopter parent stated that only one of his five children had received the COVID-19 vaccine’s initial two doses to attend a school trip, but “he hasn’t had any boosters” subsequently due to the absence of other mandates to do so (48241424).

**Figure 1:**
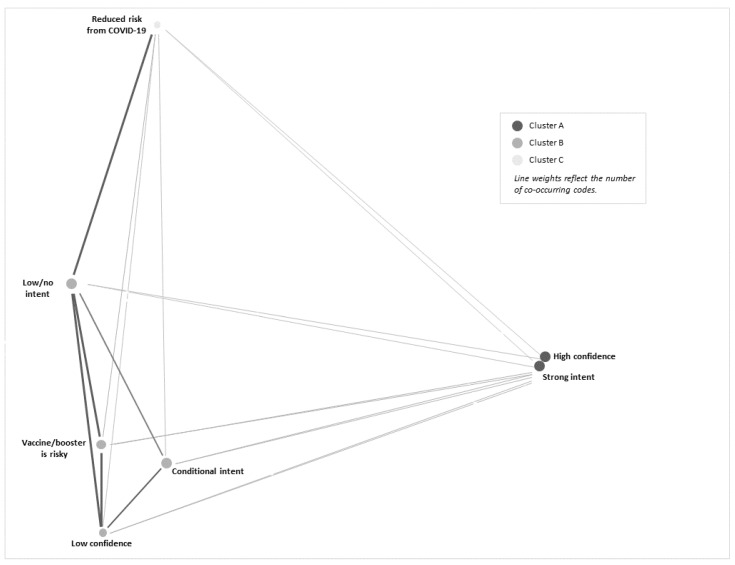
Cluster plot of secondary themes.

Risk assessments of COVID-19, confidence in the COVID-19 vaccine, and intent to vaccinate were frequently bundled by participants in their narrative for future COVID-19 vaccination behavior for their child(ren). A heatmap demonstrates how statements of high confidence in the COVID-19 vaccines by hesitant adopter parents were frequently linked to a strong intent to have their child(ren) receive future COVID-19 vaccine boosters (see Table 3). However, participants with low confidence in COVID-19 vaccines and boosters often expressed conditional and/or low/no intent to have their child(ren) receive COVID-19 boosters. The most frequently co-occurring coded segments were high confidence and strong intent to have their child(ren) receive the COVID-19 booster (co-occurring 134 times across all transcripts). The second most frequently co-occurring codes were vaccine/booster is risky and low/no intent, co-occurring 78 times. Participants’ statements were occasionally mixed or variable in their reported intent to vaccinate, with segments coded as strong intent sometimes co-occurring with segments coded as conditional and/or low/no intent (co-occurring 32 times across all interviews). We generated a cluster plot (Figure 1) of subthemes to highlight these findings; this figure demonstrates associations between high confidence and strong intent, perceived risk from the COVID-19 vaccine and low confidence with conditional intent and low/no intent, and perceptions of reduced risk from COVID-19 infection with low/no intent to have children receive COVID-19 boosters.

## 4. Discussion

This study explored hesitant adopter parents’ intentions to have their child(ren) receive a COVID-19 booster. Hesitant adopter parents’ concerns about the risks of COVID-19 vaccines and perceived risks from COVID-19 infection were critical for their intent to have their child(ren) receive COVID-19 vaccine boosters. Perceived risks from COVID-19 vaccines and low motivation were co-occurring themes with conditional and low/no intent to vaccinate, and there was a frequent co-occurrence of high confidence and strong intent to vaccinate. We found that past COVID-19 infection was commonly reported among participants and their child(ren), indicating that personal experience may contribute to participants’ perceptions of the risks of COVID-19 infection, as has been found in prior studies [47,48,49,50,51,52]. This is consistent with the broader literature on risk evaluation in the vaccination process, as well as the growing literature on COVID-19 booster acceptance [7,29,53,54]. We found hesitant adopter parents’ initial perceptions of the COVID-19 vaccine as risky remained salient for their future vaccination decisions for their children, despite previously choosing to vaccinate their child(ren). This is the first qualitative study to demonstrate that COVID-19 vaccine hesitancy among parents can persist and impact the intent to receive boosters. While further research is needed, understanding latent or persistent concerns and fears surrounding vaccines is critical for increasing COVID-19 booster uptake, for mitigating the continuing effects of COVID-19, and for the uptake of other vaccines in the future.

Some hesitant adopter parents reported high confidence in the COVID-19 vaccines, which was frequently linked to a strong intent to have their child(ren) receive future COVID-19 boosters. However, other parents described their confidence in COVID-19 vaccines and boosters as low, often linked to conditional and/or low/no intent to have their child(ren) receive COVID-19 boosters. Participants with low confidence frequently attributed this to distrust of pharmaceutical companies, the government, and public health officials; high perceived risk of side effects from the vaccine; lack of vaccine efficacy in preventing infection and transmission of COVID-19; or a combination of these factors. This is consistent with the literature in which high confidence, intent to vaccinate, and vaccination behavior are strongly associated [55,56,57]. This is the first article to confirm these associations for hesitant adopter parents in their intent to have their child(ren) receive COVID-19 boosters.

Hesitant adopter parents described their intent to vaccinate as a dynamic and ongoing process. These findings contrast with those of the prior literature that demonstrated a strong correlation between vaccination status (e.g., prior vaccination behavior) and subsequent vaccine uptake [49,51,57,58,59,60,61]. However, the findings are consistent with the literature which posits vaccination decisions as “dynamic, fluid, and often ambivalent processes” [31]. Our findings extend the current literature on vaccination-as-process and call into question the utility of prior vaccination as a predictor of future vaccination behavior for hesitant adopters. Further investigation is needed to explore the correlation between prior vaccination and intent for future vaccination, especially in light of recent research demonstrating the potential spillover of COVID-19 vaccine hesitancy to other vaccines [55] and the importance of COVID-19 vaccine boosters as tools to mitigate the ongoing effects of COVID-19 [7,8,29,53]. Processual approaches, such as Anderson’s health-as-process [62,63], Becker’s early sociological work on marijuana use [64,65], Biehl and Locke’s anthropology of becoming [66], or Chrisman’s “health-seeking process” [67], have been underutilized in understanding the dynamic nature of vaccine hesitancy [10,31]. Understanding vaccine hesitancy and vaccine uptake as a dynamic process raises pragmatic issues for interventions to improve the uptake of COVID-19 boosters and other vaccines that require multiple doses, such as the human papillomavirus vaccine [29]. Understanding vaccination as a process could improve the operationalization of vaccine hesitancy and related constructs [14] and open new opportunities in monitoring and responding to vaccine hesitancy [31,68,69].

Consistent with prior studies, participants identified provider recommendations as an important influence on their intent [61,70,71,72]. Participants also identified the lifting of mandates as affecting their intent to seek boosters, noting that many policies which facilitated vaccination had ended, increasing the effort needed to have their children vaccinated. This is consistent with prior research demonstrating the positive effects of employer policies, mandates, financial incentives, and so-called nudges on COVID-19 vaccine uptake [73,74,75]. Our findings imply that the reduced facilitation of COVID-19 vaccination can have a deleterious effect on vaccine uptake, even among those with strong intention to seek COVID-19 boosters. As Brewer and colleagues have noted, the most reliably effective interventions to increase vaccine uptake involve reducing practical barriers to vaccination; however little attention has been paid to the effects of the de-implementation of facilitating policies, mandates, incentives, etc., on vaccine uptake.

## 5. Recommendations for Practice and Directions for Further Research

Our findings highlight important issues for healthcare providers and public health officials. As prior vaccination behavior may not be a reliable indicator of future vaccine acceptance among hesitant adopters, and feelings of hesitancy can persist and drive the delay or refusal of future vaccination, healthcare providers and public health officials should continue to address parental concerns and provide strong evidence for vaccine safety and efficacy, without assuming that behaviors or feelings toward vaccines will be unchanging [30]. With the dynamic, ongoing nature of vaccination decision making, healthcare providers should continue to build provider–patient trust with parents to improve COVID-19 vaccine booster uptake [16,76,77]. This is especially critical in light of strong evidence in the literature of the role of trust in the vaccination process and health-seeking behaviors more generally, with studies showing a positive correlation between medical adherence among patients and their reported trust in physicians [72,78,79,80,81,82,83]. Globally, the importance of increasing vaccine confidence and trust in state-sponsored vaccination campaigns has been identified as a critical challenge in improving vaccine coverage [78]. Future research should further explore the vaccination process across the vaccine-hesitancy continuum and should identify causal links among social processes related to vaccine attitudes and behaviors and real-world vaccination behavior. Further research is also needed to develop novel interventions or adapt extant interventions to more effectively drive vaccine uptake [56].

## 6. Limitations and Strengths

This study is not without some limitations. Due to the focused study sample (e.g., COVID-19 vaccine-hesitant adopter parents in Arkansas), our findings may not generalize to other contexts. The primary objective of qualitative research is to explore and understand constructs and concepts by using nuanced, lived experiences of study participants which can provide trustworthy insight into their social context [84,85]. Our sample of hesitant adopter parents reported a higher level of education than Arkansas as a whole, which may affect the transferability or utility of our findings for individuals with average or lower levels of educational attainment. More than half of the participants in this study reported holding some form of post-high school degree compared to only 24.3% of Arkansans who report holding a bachelor’s degree or higher. Despite these limitations, this article makes an important contribution to the literature. To our knowledge, this is the first qualitative article to examine the role of vaccine hesitancy in influencing intent to have children receive COVID-19 vaccine boosters.

## 7. Conclusions

This paper explores the attitudes about COVID-19 vaccines and boosters and the relationship between these factors and parental intention to have their child(ren) receive COVID-19 vaccine boosters among parents in Arkansas who accepted a prior COVID-19 vaccination for their child(ren) but were hesitant to do so. Our findings address critical gaps in the understanding of vaccine hesitancy as co-occurring with vaccine uptake, especially the role of persistent parental vaccine hesitancy on intentions to have their child(ren) receive a booster. Hesitant adopter parents described their intentions to vaccinate as part of a dynamic, ongoing process and not a fixed stance towards the COVID-19 vaccine. This is the first qualitative study to explore how past attitudes of vaccine hesitancy can be salient for uptake for COVID-19 boosters. The findings of this study also call into question the reliability of prior vaccination as a predictor for future vaccine uptake, although more research is needed to examine how prior vaccination decisions may be revisited for subsequent recommended doses as the context of vaccination changes. A key finding of this study is the association of persistent concerns about the COVID-19 vaccine and low confidence in the vaccine with conditional intent, low/no intent, or refusal to receive recommended booster doses for children. Our findings have implications for healthcare providers and public health officials. Because prior vaccination behavior may not be a reliable indicator of future vaccine acceptance among hesitant adopter parents, healthcare providers and public health officials must continue to address parental concerns and provide strong evidence for vaccine safety and efficacy. This is especially critical due to the risk of severe COVID-19 outcomes for children and adolescents, booster fatigue, and low rates of COVID-19 vaccine booster uptake, even among those who have completed the primary series.

## Figures and Tables

**Table 1:** Descriptive statistics of study participants (n = 20).

	Freq.	Col. %	Mean	Range
**Age**		--	43.45	30–73
**Gender**				
Man	7	35.0		
Woman	13	65.0		
**Race/Ethnicity**				
Black/african american	4	20.0		
Hispanic	2	10.0		
Multiracial/other	1	5.0		
White	13	65.0		
**Relationship Status**				
Married	13	65.0		
Divorced	3	15.0		
Widowed	1	5.0		
Never married	3	15.0		
**Education**				
Hs grad or equivalent	4	20.0		
Some college, no degree	2	10.0		
Associate degree (aa, as)	4	20.0		
Bachelor’s degree (ba, bs, ab)	5	25.0		
Graduate degree (ma, prof, phd)	5	25.0		
**Healthcare Coverage**				
Yes	19	95.0		
No	1	5.0		
**COVID-19 Vaccination Status-Children**				
Yes, some but not all	5	25.0		
Yes, all	15	75.0		
**COVID-19 Vaccine Hesitancy**				
A little hesitant	11	55.0		
Somewhat hesitant	1	5.0		
Very hesitant	8	40.0		
**Trust in COVID-19 Vaccines**				
Not at all	2	10.0		
A little	5	25.0		
Somewhat	5	25.0		
Very much	8	40.0		
**Past/Current COVID-19 Infection—Parent**				
No	7	35.0		
Yes	13	65.0		
**Past/Current COVID-19 Infection—Child**				
No	3	15.0		
Yes, tested positive	15	75.0		
Yes, suspected	2	10.0		
**COVID-19 Death Exposure**				
No	13	65.0		
Yes	7	35.0		

**Table 2:** Qualitative themes.

Primary Themes	Subthemes
**Risk**	Reduced risk from COVID-19 infection
	Vaccine/booster is risky
**Confidence**	Low confidence
	High confidence
**Intent**	Strong intent
	Conditional intent
	Low/no intent

**Table 3:** Heatmap of theme co-occurrence.

Themes	Reduced Risk from COVID-19 *	Vaccine/Booster Is Risky	High Confidence	Low Confidence	Strong Intent	Low/No Intent	Conditional Intent			
Reduced risk from COVID-19		25	9	6	10	15	50			80+
Vaccine/booster is risky	25		20	58	23	55	78			60–79
High confidence	9	20		8	134	16	11			40–59
Low confidence	6	58	8		11	28	49			20–39
Strong intent	10	23	134	11		18	13			0–19
Conditional intent	15	55	16	28	18		32			
Low/no intent	50	78	11	49	13	32				

* The numbers are co-occurring codes. The color-coding reflects correlations between themes.

## Data Availability

The deidentified data underlying the results presented in this study may be made available upon reasonable request from the corresponding author, Pearl A. McElfish, at pamcelfish@uams.edu.
